# Monoclinic polymorph of 4-[(1*H*-benz­imidazol-1-yl)meth­yl]benzoic acid

**DOI:** 10.1107/S1600536811039043

**Published:** 2011-09-30

**Authors:** Hai-Wei Kuai, Xiao-Chun Cheng

**Affiliations:** a Faculty of Life Science and Chemical Engineering, Huaiyin Institute of Technology, Huaian 223003, People’s Republic of China

## Abstract

Three polymorphs of the title compound, C_15_H_12_N_2_O_2_, were obtained accidentally as single crystals in the hydro­thermal reaction of the title compound with manganese bromide in the presence of *N*,*N*′-dimethyl­formamide at 373 K. Here we report the structure of the first polymorph. The benzimidazole ring is almost planar, the maximum deviation from the mean plane being 0.016 (1) Å. The benzimidazole and benzene rings are approximately perpendicular, making a dihedral angle 85.56 (7)°, which is a reflection of the axial rotation of the flexible benzimidazolyl arm. In the crystal, adjacent mol­ecules are connected through O—H⋯N hydrogen bonds into a chain along [100], and neighboring chains are further linked by *via* weak C—H⋯O hydrogen-bonding inter­actions, forming a two-dimensional network.

## Related literature

For two other polymorphs of the title compound, see Kuai & Cheng (2011*a* ▶,*b* ▶). For the synthesis of 4-[(1*H*-benzo[*d*]imidazol-1-yl)meth­yl]benzoic acid, see: Hua *et al.* (2010 ▶). For background to metal-organic hybrid materials, see: Das & Bharadwaj (2009 ▶); Kuai *et al.* (2011 ▶).
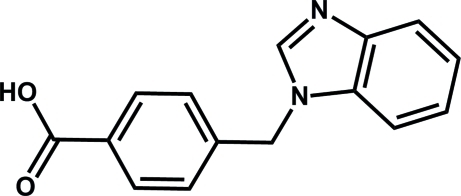


## Experimental

### 

#### Crystal data

C_15_H_12_N_2_O_2_
*M* *_r_* = 252.27
Monoclinic, 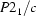

*a* = 10.435 (2) Å
*b* = 14.360 (3) Å
*c* = 8.2922 (17) Å
β = 96.925 (3)°
*V* = 1233.5 (4) Å^3^
*Z* = 4
Mo *K*α radiation
μ = 0.09 mm^−1^
*T* = 293 K
0.20 × 0.20 × 0.16 mm

#### Data collection

Bruker SMART APEX CCD diffractometer
Absorption correction: multi-scan (*SADABS*; Sheldrick, 1996 ▶) *T*_min_ = 0.982, *T*_max_ = 0.985
6155 measured reflections
2157 independent reflections
1294 reflections with *I* > 2σ(*I*)
*R*_int_ = 0.065

#### Refinement

*R*[*F* ^2^ > 2σ(*F* ^2^)] = 0.041
*wR*(*F* ^2^) = 0.082
*S* = 0.82
2157 reflections
160 parameters
H-atom parameters constrained
Δρ_max_ = 0.13 e Å^−3^
Δρ_min_ = −0.19 e Å^−3^

### 

Data collection: *SMART* (Bruker, 1998 ▶); cell refinement: *SAINT* (Bruker, 1998 ▶); data reduction: *SAINT*; program(s) used to solve structure: *SHELXS97* (Sheldrick, 2008 ▶); program(s) used to refine structure: *SHELXL97* (Sheldrick, 2008 ▶); molecular graphics: *SHELXTL* (Sheldrick, 2008 ▶); software used to prepare material for publication: *SHELXTL*.

## Supplementary Material

Crystal structure: contains datablock(s) I, global. DOI: 10.1107/S1600536811039043/aa2023sup1.cif

Supplementary material file. DOI: 10.1107/S1600536811039043/aa2023Isup2.cdx

Structure factors: contains datablock(s) I. DOI: 10.1107/S1600536811039043/aa2023Isup3.hkl

Supplementary material file. DOI: 10.1107/S1600536811039043/aa2023Isup4.cml

Additional supplementary materials:  crystallographic information; 3D view; checkCIF report

## Figures and Tables

**Table 1:** Hydrogen-bond geometry (Å, °)

*D*—H⋯*A*	*D*—H	H⋯*A*	*D*⋯*A*	*D*—H⋯*A*
O1—H12⋯N12^i^	0.82	1.83	2.652 (2)	178
C12—H7⋯O2^ii^	0.93	2.49	3.213 (3)	135

Symmetry codes: (i) 
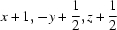
; (ii) 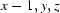.

**Fig. 1.**
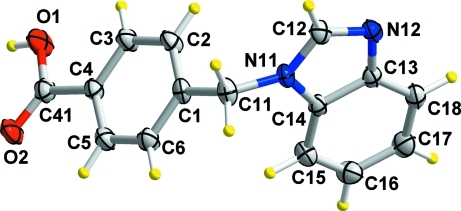
: The crystal structure of the title compound showing 30% probability displacement ellipsoids.

**Fig. 2.**
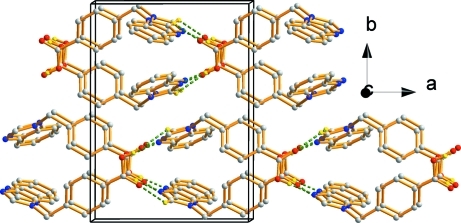
: The packing diagram of the title compound. Hydrogen bonds are shown as dashed lines.

## supplementary crystallographic information

### Comment

The title compound, 4-((1H-benzo[d]imidazol-1-yl)methyl)benzoic acid (HL), are usually regarded as an excellent candidate for building block in molecular self-assembly engineerings due to its variable conformations and coordination modes (Das *et al.*, 2009). During assemblies of coordination polymers, we accidentally obtained three different kind of single crystals of the title compound, which can be proved by different unit cell parameters (or space group). Here, we will introduce one of them. The single crystals of the title compound, C_15_H_12_N_2_O_2_, were accidentally obtained by the hydrothermal reaction at 373 K of the HL with manganese bromide in the presence of N,N'-dimethylformamide as alkaline medium for the deprotonation. As shown in Fig. 1, the asymmetric unit consists of only one HL molecule. Interestingly, though crystallizing from alkaline solution, the HL remains the intact carboxylic group in the crystal structure. The flexible benzimidazolyl arm is apt to axially rotate. as a result, the benzimidazolyl ring and central benzene rings are approximately vertical, inclined at a dihedral angle of 85.56 (6) °; The torsion angles of N11-C11-C1-C2 and N11-C11-C1-C6 are -56.9 (3) ° and 125.4 (2) °, respectively. Adjacent molecules are connected through O-H···N hydrogen bonds into a one-dimensional chain along [100] deirction, and neighboring chains are further linked via C-H···O weak hydrogen bonding interaction to form a two-dimensional network (Fig. 2).

### Experimental

Reaction mixture of MnBr_2_ (21.5 mg, 0.1 mmol), 4-((1*H*-benzo[*d*]imidazol-1-yl)methyl)benzoic acid (25.2 mg, 0.1 mmol) and 2 ml *N*,*N*'-dimethylformamide (DMF) in 8 ml H_2_O were sealed in a 16 ml Teflon-lined stainless steel container and keeped at 373 K for 3 days. After cooling to the room temperature, colourless block crystals of the title compound were obtained.

### Refinement

All hydrogen atoms were located in geometrically idealized positions and constrained to ride on their parent atoms, with C—H = 0.93 and 0.97 Å, O—H = 0.82 Å and *U*_iso_(H) = 1.2*U*_eq_(C, O).

### Crystal data

**Table:**

C_15_H_12_N_2_O_2_	*F*(000) = 528
*M**_r_* = 252.27	*D*_x_ = 1.358 Mg m^−^^3^
Monoclinic, *P*2_1_/*c*	Mo *K*α radiation, λ = 0.71073 Å
Hall symbol: -P 2ybc	Cell parameters from 961 reflections
*a* = 10.435 (2) Å	θ = 2.8–20.1°
*b* = 14.360 (3) Å	µ = 0.09 mm^−^^1^
*c* = 8.2922 (17) Å	*T* = 293 K
β = 96.925 (3)°	Block, colourless
*V* = 1233.5 (4) Å^3^	0.20 × 0.20 × 0.16 mm
*Z* = 4	

### Data collection

**Table:**

Bruker SMART APEX CCD diffractometer	2157 independent reflections
Radiation source: fine-focus sealed tube	1294 reflections with *I* > 2σ(*I*)
graphite	*R*_int_ = 0.065
phi and ω scans	θ_max_ = 25.0°, θ_min_ = 2.0°
Absorption correction: multi-scan (*SADABS*; Sheldrick, 1996)	*h* = −12→12
*T*_min_ = 0.982, *T*_max_ = 0.985	*k* = −17→14
6155 measured reflections	*l* = −9→9

### Refinement

**Table:**

Refinement on *F*^2^	Primary atom site location: structure-invariant direct methods
Least-squares matrix: full	Secondary atom site location: difference Fourier map
*R*[*F*^2^ > 2σ(*F*^2^)] = 0.041	Hydrogen site location: inferred from neighbouring sites
*wR*(*F*^2^) = 0.082	H-atom parameters constrained
*S* = 0.82	*w* = 1/[σ^2^(*F*_o_^2^) + (0.0242*P*)^2^] where *P* = (*F*_o_^2^ + 2*F*_c_^2^)/3
2157 reflections	(Δ/σ)_max_ < 0.001
160 parameters	Δρ_max_ = 0.13 e Å^−^^3^
0 restraints	Δρ_min_ = −0.19 e Å^−^^3^

### Special details

**Table:**

Geometry. All e.s.d.'s (except the e.s.d. in the dihedral angle between two l.s. planes) are estimated using the full covariance matrix. The cell e.s.d.'s are taken into account individually in the estimation of e.s.d.'s in distances, angles and torsion angles; correlations between e.s.d.'s in cell parameters are only used when they are defined by crystal symmetry. An approximate (isotropic) treatment of cell e.s.d.'s is used for estimating e.s.d.'s involving l.s. planes.
Refinement. Refinement of *F*^2^ against ALL reflections. The weighted *R*-factor *wR* and goodness of fit *S* are based on *F*^2^, conventional *R*-factors *R* are based on *F*, with *F* set to zero for negative *F*^2^. The threshold expression of *F*^2^ > σ(*F*^2^) is used only for calculating *R*-factors(gt) *etc*. and is not relevant to the choice of reflections for refinement. *R*-factors based on *F*^2^ are statistically about twice as large as those based on *F*, and *R*- factors based on ALL data will be even larger.

### Fractional atomic coordinates and isotropic or equivalent isotropic displacement parameters (Å^2^)

**Table:**

	*x*	*y*	*z*	*U*_iso_*/*U*_eq_	
N11	0.62238 (13)	0.42913 (9)	0.16767 (19)	0.0384 (4)	
N12	0.43462 (14)	0.36669 (10)	0.06416 (19)	0.043	
O2	1.30575 (12)	0.33153 (9)	0.43647 (18)	0.062	
O1	1.19958 (12)	0.20370 (10)	0.49287 (19)	0.0694 (5)	
H12	1.2729	0.1834	0.5165	0.083*	
C14	0.63655 (16)	0.40473 (11)	0.0100 (2)	0.0362 (5)	
C4	1.07691 (16)	0.33415 (13)	0.3955 (2)	0.0406 (5)	
C15	0.73758 (18)	0.41493 (12)	−0.0828 (3)	0.0479 (5)	
H8	0.8156	0.4417	−0.0406	0.057*	
C13	0.51859 (17)	0.36548 (12)	−0.0533 (2)	0.0383 (5)	
C2	0.84621 (17)	0.33703 (13)	0.3785 (2)	0.0473 (5)	
H1	0.7693	0.3075	0.3939	0.057*	
C11	0.71733 (16)	0.47882 (13)	0.2797 (2)	0.0469 (5)	
H6	0.6815	0.4900	0.3806	0.056*	
H5	0.7347	0.5388	0.2333	0.056*	
C1	0.84294 (16)	0.42581 (13)	0.3160 (2)	0.0402 (5)	
C41	1.20563 (18)	0.28995 (15)	0.4421 (2)	0.0468 (5)	
C12	0.50053 (17)	0.40470 (12)	0.1920 (2)	0.0445 (5)	
H7	0.4671	0.4140	0.2898	0.053*	
C5	1.07310 (18)	0.42162 (14)	0.3297 (3)	0.0515 (6)	
H3	1.1497	0.4503	0.3106	0.062*	
C18	0.49941 (19)	0.33416 (13)	−0.2123 (3)	0.0488 (5)	
H11	0.4214	0.3075	−0.2550	0.059*	
C6	0.95772 (18)	0.46810 (14)	0.2912 (3)	0.0523 (6)	
H4	0.9573	0.5280	0.2484	0.063*	
C17	0.5994 (2)	0.34376 (13)	−0.3053 (3)	0.0563 (6)	
H10	0.5888	0.3235	−0.4126	0.068*	
C3	0.96226 (17)	0.29079 (13)	0.4188 (2)	0.0506 (6)	
H2	0.9630	0.2309	0.4614	0.061*	
C16	0.7167 (2)	0.38356 (13)	−0.2405 (3)	0.0557 (6)	
H9	0.7827	0.3890	−0.3060	0.067*	

### Atomic displacement parameters (Å^2^)

**Table:**

	*U*^11^	*U*^22^	*U*^33^	*U*^12^	*U*^13^	*U*^23^
N11	0.0279 (9)	0.0422 (10)	0.0433 (11)	0.0015 (7)	−0.0023 (8)	−0.0023 (8)
N12	0.032	0.044	0.053	−0.002	−0.001	−0.001
O2	0.029	0.070	0.085	−0.006	0.001	0.011
O1	0.0326 (8)	0.0644 (11)	0.1098 (14)	0.0077 (7)	0.0023 (8)	0.0228 (9)
C14	0.0324 (11)	0.0324 (11)	0.0427 (13)	0.0008 (8)	−0.0007 (9)	0.0012 (9)
C4	0.0299 (11)	0.0493 (13)	0.0418 (13)	0.0004 (9)	0.0014 (9)	0.0007 (10)
C15	0.0392 (12)	0.0494 (13)	0.0543 (15)	−0.0065 (10)	0.0027 (11)	−0.0049 (11)
C13	0.0350 (11)	0.0304 (11)	0.0474 (13)	0.0020 (8)	−0.0033 (10)	0.0027 (10)
C2	0.0282 (11)	0.0524 (14)	0.0612 (15)	−0.0028 (9)	0.0046 (10)	−0.0005 (11)
C11	0.0361 (12)	0.0481 (13)	0.0539 (14)	0.0039 (9)	−0.0054 (10)	−0.0101 (10)
C1	0.0323 (11)	0.0425 (13)	0.0435 (13)	0.0008 (9)	−0.0046 (9)	−0.0076 (10)
C41	0.0355 (12)	0.0548 (14)	0.0494 (14)	0.0029 (10)	0.0021 (10)	0.0018 (11)
C12	0.0339 (11)	0.0472 (12)	0.0523 (14)	0.0060 (9)	0.0048 (10)	0.0027 (10)
C5	0.0338 (12)	0.0566 (14)	0.0634 (15)	−0.0062 (10)	0.0022 (11)	0.0068 (12)
C18	0.0425 (12)	0.0451 (13)	0.0553 (15)	−0.0032 (10)	−0.0078 (11)	−0.0021 (11)
C6	0.0415 (13)	0.0460 (13)	0.0668 (16)	−0.0013 (10)	−0.0032 (11)	0.0092 (11)
C17	0.0699 (16)	0.0486 (14)	0.0492 (15)	−0.0045 (11)	0.0024 (13)	−0.0046 (11)
C3	0.0354 (12)	0.0473 (13)	0.0683 (16)	0.0006 (10)	0.0035 (11)	0.0103 (11)
C16	0.0605 (15)	0.0514 (14)	0.0574 (16)	−0.0101 (11)	0.0164 (13)	−0.0028 (12)

### Geometric parameters (Å, °)

**Table:**

N11—C12	1.357 (2)	C2—C3	1.386 (2)
N11—C14	1.379 (2)	C2—H1	0.9300
N11—C11	1.460 (2)	C11—C1	1.514 (2)
N12—C12	1.311 (2)	C11—H6	0.9700
N12—C13	1.387 (2)	C11—H5	0.9700
O2—C41	1.209 (2)	C1—C6	1.380 (2)
O1—C41	1.312 (2)	C12—H7	0.9300
O1—H12	0.8200	C5—C6	1.380 (2)
C14—C15	1.386 (2)	C5—H3	0.9300
C14—C13	1.397 (2)	C18—C17	1.377 (2)
C4—C5	1.368 (2)	C18—H11	0.9300
C4—C3	1.383 (2)	C6—H4	0.9300
C4—C41	1.494 (2)	C17—C16	1.398 (3)
C15—C16	1.375 (3)	C17—H10	0.9300
C15—H8	0.9300	C3—H2	0.9300
C13—C18	1.384 (3)	C16—H9	0.9300
C2—C1	1.375 (2)		
			
C12—N11—C14	106.55 (15)	C2—C1—C11	121.50 (16)
C12—N11—C11	127.41 (16)	C6—C1—C11	119.67 (18)
C14—N11—C11	125.84 (15)	O2—C41—O1	123.63 (18)
C12—N12—C13	105.01 (15)	O2—C41—C4	122.30 (19)
C41—O1—H12	109.5	O1—C41—C4	114.05 (16)
N11—C14—C15	132.49 (17)	N12—C12—N11	113.38 (17)
N11—C14—C13	105.55 (15)	N12—C12—H7	123.3
C15—C14—C13	121.93 (18)	N11—C12—H7	123.3
C5—C4—C3	119.02 (17)	C4—C5—C6	121.26 (17)
C5—C4—C41	118.38 (17)	C4—C5—H3	119.4
C3—C4—C41	122.59 (18)	C6—C5—H3	119.4
C16—C15—C14	116.72 (19)	C17—C18—C13	117.92 (19)
C16—C15—H8	121.6	C17—C18—H11	121.0
C14—C15—H8	121.6	C13—C18—H11	121.0
C18—C13—N12	129.94 (18)	C5—C6—C1	120.08 (19)
C18—C13—C14	120.54 (18)	C5—C6—H4	120.0
N12—C13—C14	109.51 (17)	C1—C6—H4	120.0
C1—C2—C3	121.09 (17)	C18—C17—C16	120.9 (2)
C1—C2—H1	119.5	C18—C17—H10	119.6
C3—C2—H1	119.5	C16—C17—H10	119.6
N11—C11—C1	112.67 (15)	C4—C3—C2	119.73 (18)
N11—C11—H6	109.1	C4—C3—H2	120.1
C1—C11—H6	109.1	C2—C3—H2	120.1
N11—C11—H5	109.1	C15—C16—C17	121.99 (19)
C1—C11—H5	109.1	C15—C16—H9	119.0
H6—C11—H5	107.8	C17—C16—H9	119.0
C2—C1—C6	118.79 (17)		
			
C12—N11—C14—C15	178.01 (19)	C3—C4—C41—O2	−172.53 (19)
C11—N11—C14—C15	2.8 (3)	C5—C4—C41—O1	−175.23 (18)
C12—N11—C14—C13	−0.18 (18)	C3—C4—C41—O1	5.9 (3)
C11—N11—C14—C13	−175.40 (16)	C13—N12—C12—N11	0.2 (2)
N11—C14—C15—C16	−178.63 (19)	C14—N11—C12—N12	0.0 (2)
C13—C14—C15—C16	−0.7 (3)	C11—N11—C12—N12	175.12 (17)
C12—N12—C13—C18	−179.09 (19)	C3—C4—C5—C6	2.0 (3)
C12—N12—C13—C14	−0.3 (2)	C41—C4—C5—C6	−176.89 (19)
N11—C14—C13—C18	179.22 (16)	N12—C13—C18—C17	178.11 (18)
C15—C14—C13—C18	0.8 (3)	C14—C13—C18—C17	−0.6 (3)
N11—C14—C13—N12	0.3 (2)	C4—C5—C6—C1	−1.2 (3)
C15—C14—C13—N12	−178.13 (16)	C2—C1—C6—C5	−0.4 (3)
C12—N11—C11—C1	124.04 (18)	C11—C1—C6—C5	177.53 (19)
C14—N11—C11—C1	−61.7 (2)	C13—C18—C17—C16	0.3 (3)
C3—C2—C1—C6	1.1 (3)	C5—C4—C3—C2	−1.3 (3)
C3—C2—C1—C11	−176.76 (18)	C41—C4—C3—C2	177.59 (18)
N11—C11—C1—C2	−56.8 (2)	C1—C2—C3—C4	−0.3 (3)
N11—C11—C1—C6	125.34 (19)	C14—C15—C16—C17	0.4 (3)
C5—C4—C41—O2	6.3 (3)	C18—C17—C16—C15	−0.2 (3)

### Hydrogen-bond geometry (Å, °)

**Table:**

*D*—H···*A*	*D*—H	H···*A*	*D*···*A*	*D*—H···*A*
O1—H12···N12^i^	0.82	1.83	2.652 (2)	178.
C12—H7···O2^ii^	0.93	2.49	3.213 (3)	135.

Symmetry codes: (i) *x*+1, −*y*+1/2, *z*+1/2; (ii) *x*−1, *y*, *z*.
